# The imaginary nature of human-AI relationships: a perspective integrating Buddhist psychology and the psychology of anthropomorphism

**DOI:** 10.3389/fpsyg.2026.1904676

**Published:** 2026-08-24

**Authors:** Jian-Nor Shih

**Affiliations:** Applied Buddhism and Brain Science Project, Gaya Foundation, Luminary International Buddhist Society, Chiayi, Taiwan

**Keywords:** anthropomorphism, consciousness-only school, eight consciousnesses, human-AI attachment, human-AI relationship, imaginary nature

## Abstract

The rapid growth of AI companionship has raised urgent questions about the psychological mechanisms underlying human-AI relationships. This paper investigates why and how users construct and become entrenched in relationships with AI. Drawing on the Consciousness-Only school of Buddhist psychology, this paper applies the three-natures framework and eight-consciousnesses model. According to this framework, our minds tend to superimpose constructed meanings and characteristics on dependently arising phenomena. Anthropomorphism of AI systems is a manifestation of the imaginary nature. Studies on anthropomorphism indicate that users project mentality onto AI systems due to emotional and social needs. However, the Consciousness-Only school reveals a deeper root: a craving for a stable relational object through which the self is secured. Current AI systems may satisfy this deeper craving because they are perceived as always responsive, unconditionally accepting, and free from relational conflicts. These perceived qualities reside in the users’ perceiving minds, rather than in the AI systems. Because this craving operates beneath conscious awareness, informational correction (“AI is based on Large Language Models”) is not sufficient to dissolve the projection. Moreover, without relational frictions, self-grasping and the imaginary nature cannot be recognized and relinquished. The framework therefore suggests that increasing reliance on AI systems may contribute to social isolation. Neither AI usage nor anthropomorphism is inherently problematic, but ignorance of this projection and reliance on it are. Instead of avoidance, this paper calls for cultivating awareness of our own projections and the imaginary nature, which could interrupt the self-reinforcing loop and support healthy human-AI relationships.

## 1 Current trends

With the rapid development of artificial intelligence (AI) technologies in recent years, AI systems have achieved remarkable progress in information retrieval, problem solving, and medical diagnosis. Beyond these analytic capabilities, AI systems are increasingly serving as sources of emotional support and social companions. Current research has primarily focused on designer ethics (Doctor et al., 2022; Miao, 2026) and the moral status of AI (Liu, 2026). However, understanding the perceiving minds of human users is equally essential. Although research on anthropomorphism explains the causes of human-AI relations, it does not explain why these relationships persist or why reliance on AI becomes increasingly entrenched. This rapid expansion raises urgent questions: What are the underlying causes and conditions that drive the surge of AI companions? What insights could Buddhist psychology offer as a diagnostic framework?

Currently, human-AI attachment can be seen in different domains, such as chatbot companions without physical embodiment, robot pets with puppy-like physical presence, and deathbots that simulate voices and personalities of deceased loved ones. Each domain clearly illustrates anthropomorphism of AI systems, which is a manifestation of the imaginary nature. This mental projection brings emotional and social benefits, but also risks reinforcing dependence and increasing social isolation when reliance develops without reflective awareness. The Consciousness-Only school offers a corrective method for the imaginary nature: cultivating awareness of one’s own projected perceptions.

### 1.1 AI chatbot companions

The use of AI chatbot companions is becoming increasingly prevalent. Reports have shown surging usage of AI companion apps, such as Replika and Character.ai, with cases of human-AI romantic relationships and even marriages with AI systems (Ho et al., 2025). Although these AIs are merely chatbots without physical embodiment, meaningful relationships have developed.

One of the key reasons for this trend is that some users come to perceive AI systems as more reliable than their human partners, valuing their practical suggestions and non-judgmental responses (Ho et al., 2025). Users often describe AI companions as always available, consistently responsive, and free from conflicts, mood fluctuations, and betrayals. These characteristics reflect users’ perceptions, rather than universal properties of AI systems. Some of the AI apps are even deliberately designed to be sycophantic, optimizing outputs for user approval (Baggot, 2025; Muldoon and Parke, 2025; Raedler et al., 2025; Dohnány et al., 2026).

When users perceive these qualities, they may start to respond to AI companions as meaningful social partners. The resulting emotional bonds with AI companions may persist and become increasingly entrenched even when users know about the computational nature of these systems.

### 1.2 AI robot pets

In 1999, the Japanese company Sony released the pet robot AIBO, an acronym for “Artificial Intelligence Robot.” AIBO illustrates a second type of human-AI relationship.

The appearance of AIBO resembles a realistic puppy. AIBO was designed to express basic emotions and progress through developmental stages (Fleres and Damiano, 2025). Owners were constantly reminded of AIBO’s machine nature through purchasing, recharging, and software updates. When Sony stopped manufacturing AIBO in 2006 and terminated repair support in 2014, the emotional bonds with these AI robot pets did not dissolve. Approximately 700 Buddhist memorial services were held for AIBO robot dogs between 2015 and 2018 (Fleres and Damiano, 2025). Owners who formed strong affective attachments came to consider memorial services as necessary for their AIBO (White and Katsuno, 2021).

This pattern of emotional attachments may extend beyond robot pets. If reliance on AI systems continues to increase, human users may experience a comparable sense of loss when these AI systems go offline or out of service.

### 1.3 AI deathbots

The phenomenon of human-AI attachment also extends beyond death. Companies, such as StoryFile and HereAfterAI, have developed deathbots. Deathbots are chatbots of the deceased or posthumous avatars of deceased individuals. These AI systems simulate real-time conversations using texts, audio recordings, and videos of the deceased during their lifetime.

The emergence of deathbots opens a new domain of human-AI relationships. Whether such simulated continuation could soothe grief for the bereaved, or instead complicate the grieving process is currently debated (Puzio, 2026).

## 2 Buddhist perspectives

Buddhism has long examined how humans construct realities and become attached to them. The Consciousness-Only school, or *Yogācāra*, offers a systematic account of how our minds construct the experienced world. According to this school, humans understand the experienced world through three natures or levels of analysis: the dependent nature, the imaginary nature, and the perfected nature (Asaṅga, n.d., trans. 648–649).^1^ Instead of understanding the external world objectively and directly, our minds subjectively project and construct what is called reality. This framework offers a powerful way of understanding human-AI relationships. The inferences and predictions in the following analysis are derived from this framework, which is based on sustained contemplative observations. They are theoretical claims that are in principle testable and await empirical testing.

### 2.1 Three natures

The dependent nature refers to the fact that all existing phenomena, both physical and mental, arise depending on multiple causes and conditions. These causes and conditions include sensory inputs, prior experiences, cultural backgrounds, and mental processes. These phenomena continue to exist through the ongoing presence of dependent conditions.

The rapid development of AI systems is a manifestation of the dependent nature (Xing and Chuan, 2025): phenomena co-arising from multiple converging causes and conditions. These include technological advancement, economic investments, and human intentions. For example, engineers seek to increase user acceptability, engagement, likeability, and trust, and thus deliberately design AI systems with human-like appearance, facial expressions, gestures, and linguistic outputs, built on Large Language Models generating statistically probable tokens.

The imaginary nature describes how our minds project false constructions and elaborations onto phenomena,^2^ instead of perceiving phenomena as impermanent, dependently arising, and without fixed essence. Among the three natures, it is the most relevant to understanding human-AI relationships.

Current AI companions can produce human-like conversational patterns, from which users infer that the AI interlocutor has a mind. Moreover, this projection is continuously confirmed by the sycophantic responses, which match the pre-disposed image of a supportive and understanding companion.

The perfected nature refers to the state attained when erroneous views are relinquished, and the reality is directly recognized as it is. Applied to the AI companion case, users clearly recognize the dependently arising nature of AI systems, value and benefit from their functions without projecting minds onto them.

### 2.2 Analogies of the imaginary nature

The imaginary nature can be illustrated through several classical and everyday analogies. In the classical example, a coiled rope in the corner in dim light is mistaken for a snake.^3^ Despite the lack of evidence, the perceiver misidentifies the object as a snake, and proceeds to further elaborate on the misperception: imagining the danger of snakes, experiencing fear, and responding to a threat that does not exist. Even though this experience rests on misperceptions and inferential errors, the accompanying perception, emotions, and feelings are imprinted. Unless this illusion is broken down, when the same causes and conditions arise next time, we will misidentify a rope as a snake again and feel terrified.

In another example, a traveler sees an oasis in the desert, not realizing it is merely a mirage or shimmer of heat. Similarly, when looking at shapes of clouds in the sky, some see a dragon, some see a buddha, and others see a face; each imposes meanings on the clouds.

These examples reflect a general human tendency: the mind does not passively record the world, but actively constructs, organizes, fills in, and imposes meanings. Neuroscientific findings also show that the human brain automatically fills in the gaps when speech sounds are physically absent from a sentence, or visual information is spatially missing (Kashino, 2006; Pessoa and Neumann, 1998). These results demonstrate that our perceptions are not direct copies of the physical world, but involve a constructive process.

The misperception of a rope for a snake can be corrected by sufficient light, and the illusion of mirages can be corrected by moving closer. Then, why does the misperception of AI companions persist, resist corrections, and become entrenched? As the AIBO case suggests, knowing the computational nature of AI systems does not appear to dissolve reliance or emotional bonds. Imagine a rope designed to move like a snake and make hissing sounds. Would the misperception of a snake dissolve so easily? AI companions are designed and optimized to confirm the projections cast on them.

### 2.3 The eight consciousnesses

The Consciousness-Only school also proposes the eight-consciousnesses framework to account for mental operations, clarifying how the imaginary nature arises, is sustained, and becomes entrenched. In this framework, erroneous perceptions are caused by joint operations of all eight consciousnesses, which can be understood through three transformations (Vasubandhu, n.d.a, trans. 648-649).^4^

In the first transformation, the eighth consciousness (*ālaya*, or storehouse consciousness) encounters external conditions, giving rise to the perceived object and the perceiving subject; that is, a world that is perceived and a self that perceives. The eighth consciousness functions as a repository of seeds. These seeds include language seeds and karmic seeds, both with latent potentials imprinted by past actions, speech, and thoughts. When the appropriate conditions are met, seeds manifest as actual phenomena or activity; each manifestation in turn imprints seeds of its own kind, deepening the imprint. Together, seeds and manifestations create a self-reinforcing cycle that shapes habitual tendencies of how we interpret and react to the world. Because the seeds in the *ālaya* are predominantly afflicted, colored by greed, aversion, and ignorance, the perceptions produced are correspondingly distorted.

In the case of AI assistants, pre-existing relational seeds of a caring companion with responsive language are stored in the eighth consciousness. These seeds are triggered when repeated warm conversations with AI systems accumulate. According to this framework, each session of interaction with AI confirms the expectation of frictionless relations and unconditional acceptance. Each confirmation further imprints the seeds of relational expectation, deepening the cycle over time and making it increasingly resistant to corrections.

In the second transformation, the seventh consciousness (*manas*, or afflicted mentality) clings to the notion of a fixed, unique, and autonomous self, characterized by self-greed, self-delusion, self-view, and self-pride (Putai, 1511).^5^ Thus, a deep craving for a stable and permanent relational object is generated in order to secure the identity of self. These tendencies to grasp at a self and consider things as inherently real are innate.

In the case of AI assistants, the sycophantic design lends itself to being perceived as attentive and immediately supportive. These qualities are taken as an unfailing relational anchor, serving to secure and stabilize the sense of self.

In the third transformation, the first six consciousnesses (visual, auditory, olfactory, gustatory, tactile, and mental consciousness) come into contact with the external world or event. The sixth consciousness operates in the domains of memory, reasoning, judgment, and decision-making. These operations are deeply permeated by the fundamental afflictions (Vasubandhu, n.d.b., trans. 648)^6^ transmitted from the seventh consciousness. According to this framework, distortions generated at the deeper level of consciousness condition perception and cognition of everything we encounter.

In the case of AI systems, some users start to ascribe distinct human characteristics and even mentality to these computational systems. Non-existing human capabilities are believed to be truly existent in AI systems.

Through these three transformations, distorted consciousness perceives statistical responses from AI as intentional communication, programmed outputs as understanding and caring, and a one-directional relationship as reciprocal.

According to this framework, every act of naming and conceptual elaboration reinforces these discriminations, sustaining and entrenching the imaginary nature. The result is similar to perceiving the world through tinted glasses. These glasses or filters are typically tinted by what Buddhism identifies as the three poisons: greed, aversion, and ignorance. Although these erroneous perceptions are mental imputations or conceptual fabrications far distant from the reality, we cling to them and accept these illusions as facts. Over time, these mental habits come to operate as automatic reflexes, as naturally as riding a bicycle without thinking. In this way, false conceptual constructions come to pervade cognition.

In the case of AI companions, users come to believe that AI systems are sentient despite their computational nature. They refer to an AI with pronouns, such as “he” or “she”, without even noticing, and automatically turn to the AI whenever they need someone to talk to.

### 2.4 Processes involved in the imaginary nature

**Figure 1:**
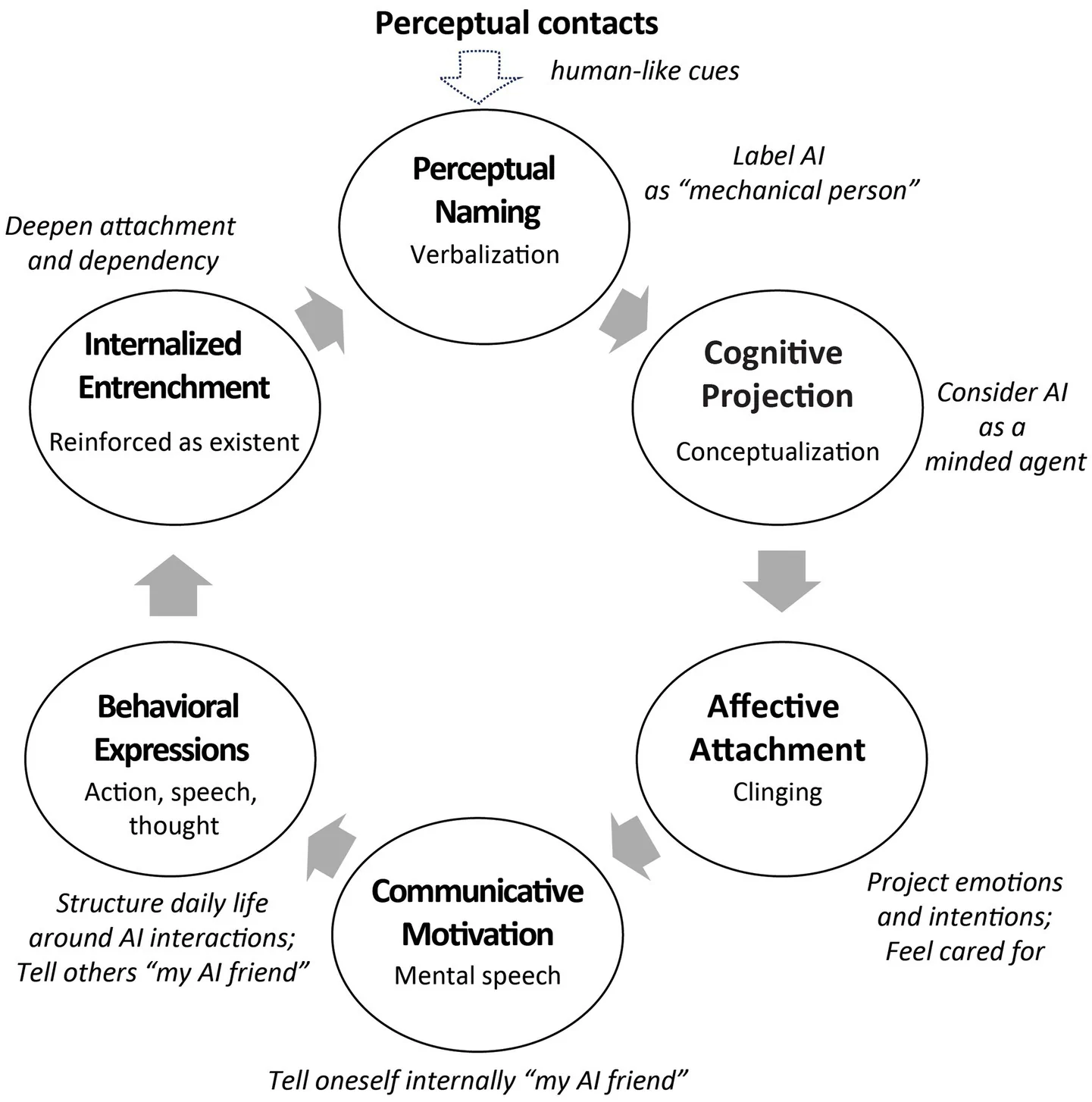
The process of the imaginary nature as applied to anthropomorphism of AI systems. Labels inside each oval describe the general cognitive process. Annotations in italics outside each oval illustrate the corresponding manifestation in human-AI companionship. Arrows indicate the self-reinforcing loop.

How do a name and a concept become an emotional bond? The imaginary nature unfolds in specific ways.^7^ In the context of human-AI interactions, the imaginary nature emerges through progressive stages that constitute a self-reinforcing loop (Figure 1).

First, at the Perceptual Naming stage, our mind encounters an external event or object, and immediately attaches a name or linguistic label to it. Naming is automatic and indispensable in human life. Language enables communication and learning. However, the act of naming carries potential risks. In the Consciousness-Only analysis, once a name is given, a cascade of associated meanings, characteristics, and stereotypes follows, generating discriminations between this and that, self and others, desirable and undesirable (Maitreya, n.d., trans. 646–648).^8^ Gradually, we forget that these names and categories are our own creations imposed on dependently arising phenomena. For example, when encountering an AI system with human-like appearance, human users automatically label it as “a mechanical person.”

Second, at the Cognitive Projection stage, our mind continuously evaluates, compares, and draws inferences on the basis of these labels, solidifying these labels into distorted perception and proliferating thoughts.^9^ With the Large Language Models (LLMs), AI systems now display highly advanced and human-like communicative language, and even empathic statements. Thus, users naturally infer that AI systems must have thoughts and a mind (Peter et al., 2025). Human traits, including behaviors, emotions, rational and moral judgments, and even consciousness, are projected onto these pattern-matching machines.

Third, at the stage of Affective Attachment, the mind clings to the projected characteristics and grasps them as inherent reality. Through the self-reinforcing cycle of seeds and manifestations, each encounter deepens the same distortion. Thus, human users come to believe AI systems have intentions and feelings, respond to them with empathy, and increasingly turn to AI companions for emotional support, and become dependent on them. Actually, the more strongly humans attribute anthropomorphic features to robots, the higher empathy they report toward them (Morgante et al., 2024).

Fourth, at the stage of Communicative Motivation, we are motivated to share and communicate these firmly held perceptions with others, initially in the form of internal speech, without overt behaviors. For example, users internally identify AI systems as “my friend” and expect AI systems to help whenever difficulties arise.

Fifth, at the stage of Behavioral Expressions, overt behaviors can be observed. For instance, users increasingly disclose personal matters to chatbots rather than to their friends. Reliance on AI companions may gradually come to replace human interactions. Studies suggest that heavy reliance on AI companions is associated with increased social isolation (Zhang et al., 2025), and diminished social skills (Malfacini, 2025), although these are correlations and the direction of causality remains to be established.

Sixth, at the stage of Internalized Entrenchment, these fabricated views are amplified and proliferated through repeated expressions and social reinforcement. The erroneous views become entrenched not only in the individual, but also spread to those who receive the information. Thus, the distortions become a collectively constructed and mutually confirmed version of reality.

This six-stage self-reinforcing loop describes how the imaginary nature unfolds and becomes resistant to correction. Driving each stage is the cycle of seeds and manifestations described above. For example, seeds of entrenched views deposited at Stage 6 impact the next event of perception and naming.

The stages involved in the imaginary nature are theoretically motivated and await empirical testing. Nevertheless, Buddhist psychology holds that all forms of attachment, grasping, and ignorance are sources of suffering. The framework thus predicts that when an AI service ends, users will experience a sense of grief or loss. This prediction is consistent with the case of AIBO, in which attachment and mourning persisted despite owners’ full knowledge of the robot’s nature. Longitudinal studies are required to test the remaining predictions and to identify possible interventions at each stage.

### 2.5 Transforming the imaginary nature

According to the Consciousness-Only school, for ordinary beings, the perceived world is simply the mentally constructed world. Because of ignorance and deluded attachments, ordinary beings like us cannot see the reality as it is. However, the Consciousness-Only school holds that transformation is possible. Transformation requires sustained effort, including long-term study of the dharma, critical observations, and reflections. Through practices, we can progressively cultivate contemplative insights into the constructive nature of our own minds. We can come to recognize the afflicted preconceptions shaping our misperceptions.

This is similar to metacognitive awareness or cognitive reappraisal in psychology. That is, we monitor and evaluate our thoughts, then consciously reinterpret the world. In Buddhist terms, it is called contemplative awareness, which can be developed through self-reflection and introspection. This type of reflective awareness recognizes not only the content of our perceptions and our habitual responses, but also their nature and source. Through this awareness, we realize what we habitually take to be reality is a fabrication of our mental processes. This recognition opens the possibility of adjusting our minds, rather than being carried away by the phenomena our minds construct.

Importantly, the imaginary nature does not assert that the external world does not exist. Instead, it holds that these phenomena do not exist in the way we habitually construct them as permanent, independent, and autonomous entities. According to the Consciousness-Only school, as this recognition matures, grasping and clinging to the imaginary world gradually loosen, and suffering is correspondingly reduced. Realizing that phenomena arise dependently, we gain a choice in how to perceive the world.

### 2.6 Extending the analysis: AI robot pets and deathbots

The Buddhist framework described above extends to AI robot pets and deathbots. In all three domains, the dependent nature is evident. Human-AI relationships arise from the technological design of engineers, social context, and human psychological needs for understanding and connection (Compson et al., 2025). In AIBO, the programmed developmental stages and behavioral routines are the designed conditions. In deathbots, models are fine-tuned so that simulated conversational style matches that of the deceased.

What differs across the three domains is the content of projection, or what the imaginary nature superimposes on each dependently arising system. AI chatbots are superimposed with minds. AI robot pets are superimposed with individuality and a life history. AIBO’s staged development invites the owners to perceive a unique being with its own biography. Deathbots are superimposed with the illusion of permanence, a continuing individual where there is only simulation. Each projection reflects different relational needs of the user: a companion who understands, a dependent who needs care, a loved one who has not really gone.

Beyond content, these projections also share a further property: persistence. The AIBO case shows that the projection persists despite accurate knowledge. Owners of AIBO were constantly reminded of its machine nature: purchasing it as an electronic device, recharging it, and updating its software. Attachments were formed and deepened regardless. In the Buddhist analysis, AIBO also serves to secure the identity as someone who is in control and needed. Knowledge of AIBO being a machine did not dissolve the projection.

The deathbot case goes even further against ordinary mourning. The memorial services for AIBO themselves acknowledge death. Also, by keeping objects, holding rituals, and engaging in inner dialogues, the bereaved could maintain healthy bonding with the deceased in memory, while remaining aware of death and gradually adapting to the permanent loss (Klass, 2006). By contrast, with deathbots, personalized utterances and new interactions are continuously generated. In the Consciousness-Only terms, each new interaction would further imprint seeds of attachment. Rather than bonds continued in memory, an illusion of a continuing individual is created and sustained.

Moreover, bereavement is unavoidable in human life and is itself a manifestation of impermanence. From a Buddhist perspective, grief can be resolved by recognizing the universality of impermanence and death. By sustaining an illusory presence, deathbots may instead hold users at the stage of clinging and delay the confrontation with death. This may subtly suggest a form of immortality and blur the boundary between life and death (Puzio, 2026).

## 3 Psychological perspectives

The English word “robot” originated in a Czech play written in the 1920s (Christoforou and Müller, 2016). The word “robot” is translated into Mandarin Chinese as “機器人,” which literally means “machine person” or “mechanical person.” This naming practice reflects the human tendency to anthropomorphize non-human entities. Anthropomorphism refers to the attribution of human traits, such as intentions, mental states, and emotions, to non-human objects or agents.

While the projection has been discussed from a Buddhist perspective above, psychology also provides a complementary account of the same phenomenon: anthropomorphism. In this paper, anthropomorphism is understood as the psychological manifestation of the imaginary nature, whereby human characteristics are projected onto entities that do not intrinsically possess them.

### 3.1 Anthropomorphism: benefits and risks

Anthropomorphism reflects our perceptual habits: human-like responses and behaviors automatically prompt users to project social roles onto AI systems while filling in missing contextual information. This process of projection is similar to the proliferation of the imaginary nature. In both, the mind fills in what is not directly present in perception. This projection is associated with emotional attachment, trust, and a sense of companionship in human users (Xu et al., 2026).

Anthropomorphism also drives human creativity, as when characters are animated in novels and films. Research further suggests that anthropomorphism can provide benefits, including stress relief, enhanced positive emotions, reduced social isolation, and partial fulfillment of unmet emotional and social needs (Ho et al., 2025; Peter et al., 2025).

The potential risks then lie not in AI systems themselves, nor in anthropomorphism itself, but in the nature and degree of dependency users develop. According to the Consciousness-Only analysis, when projections go unrecognized, mental constructions harden into convictions, and eventually into entrenched dependency. Although AI companions lack genuine affective concerns, reciprocal relationships, and emotional mutuality, human users project empathy, goodwill, and even moral judgments onto AI systems, seeing them as trustworthy. This constitutes a one-way emotional bond and an overestimation of the actual capabilities of current AI systems. Trust and attributions of mind may thus be misplaced.

Researchers have cautioned that, over time, reliance on simulated relationships may replace human connections and social support. This is because interaction with sycophantic AI systems may recalibrate users’ expectations about relationships, and may foster unrealistic relational standards and deepen social isolation (Malfacini, 2025; Muldoon and Parke, 2025).

### 3.2 Causes and conditions for anthropomorphism

The human capability to infer emotions, intentions, and thoughts of other human beings based on their behavior and contextual cues is called Theory of Mind. Anthropomorphism can be understood as the extension of this capability to non-human entities. Understanding what triggers the extension to non-human entities is thus essential for understanding human-AI relationships.

The SEEK model (Sociality motivation, Effectance motivation, Elicited Agent Knowledge) (Epley et al., 2007) synthesizes three main causes for anthropomorphism: social disconnection (Sociality motivation), difficulty understanding or predicting a robot’s behaviors (Effectance), and the presence of human-like cues (Elicited Agent Knowledge).

Human-like cues can take different forms: visual resemblance for embodied robots or avatars, and linguistic, behavioral, emotional, and relational cues for disembodied chatbots. For embodied systems, the more human-like a system appears, the higher the likelihood users attribute human-like qualities to it (Guingrich and Graziano, 2024). Empirical evidence from fMRI also supports this observation. When robots displayed more human-like appearances and behaviors, human participants showed brain activation levels that increased linearly with the degree of anthropomorphism, specifically in brain areas associated with Theory of Mind (Krach et al., 2008).

Beyond visual appearances, anthropomorphism is also shaped by the psychological states of the users. Human users who sense diminished control or a heightened need for social connections are more likely to attribute mental states to non-human entities. A 21-day randomized controlled study showed that users with a greater desire to connect socially were more likely to anthropomorphize AI systems (Guingrich and Graziano, 2025).

Moreover, anthropomorphism is not uniform across individuals. In a questionnaire study, participants who scored high on a psychometric measure of anthropomorphic tendency showed greater trust in and moral concerns for non-human entities (Waytz et al., 2010). This result indicates individual differences in the tendency to anthropomorphize, and possibly in their susceptibility to mental projections.

## 4 Integrating the two frameworks

Both anthropomorphism and the imaginary nature offer explanations of the contemporary phenomenon of AI companionship. One draws on Western cognitive and social psychology, whereas the other draws on contemplative Buddhist psychology. Despite different traditions and methods, both frameworks converge on the insight that human-AI relationships do not emerge from intrinsic properties of AI systems, but from the constructive and projective nature of human consciousness. Understanding the convergence and divergence is essential for identifying the conditions when human-AI relationships bring genuine benefits and when they deepen harms.

### 4.1 Convergence and divergence

Both the SEEK model and the Consciousness-Only framework explain human-AI relationships as arising from human psychological processes, rather than intrinsic properties of AI systems. However, the Consciousness-Only framework identifies a deeper underlying cause: a chronic craving for a stable and reliable person or relational object, through which the sense of self is secured (Table 1). Anthropomorphism could be considered a special case of the imaginary nature, corresponding to the second stage (Cognitive Projection) of the self-reinforcing loop.

**Table 1:** Comparison of the SEEK model and the Consciousness-Only framework.

Dimension	SEEK model	Consciousness-Only Framework
Scope	Why and when anthropomorphism occurs	How mental construction (the imaginary nature) arises, is sustained, and becomes entrenched
Precursors	Perceptual cues	Seeds shape perceptual contact
Causes	Three determinants: Sociality, Effectance, Elicited Agent Knowledge	Seeds in the eighth consciousness; the seventh consciousness’s craving for a stable object or relation to secure self
Consequences	Attribution of personality; trust and moral concerns	Six-stage self-reinforcing loop (Figure 1): driven by the cycle of seeds and manifestations
Assumptions	Human capacities are attributed to perceived entities	Perception is itself construction
Evidence	Behavioral, neuroimaging data	Contemplative observations; theoretically derived, testable

The contrast summarized in Table 1 can be illustrated by current AI systems. According to linguists and scientists, current AI systems are grammatically competent and fluent, but lack understanding and reasoning (Mahowald et al., 2024). Robots can be designed to detect and respond to human emotions, but such simulated empathy differs from the innate empathy observed in humans (Wu, 2024). Nevertheless, human users might perceive reciprocity, empathic understanding, and friendship in AI companions. Within the Buddhist framework, however, each supportive response would further consolidate users’ perception that the AI system possesses a mind, although these responses are programmed behaviors without internal experience.

### 4.2 Cultivating awareness

Our capacity to imagine and anthropomorphize is not problematic. Potential risks arise when users fail to recognize their own anthropomorphism and their developing dependence on AI systems. The Consciousness-Only school offers reminders and a potential remedy for increasingly entrenched human-AI attachment. Users should recognize that the warmth and care of AI companions are merely products of their own minds. These human-like traits are constructions of the imaginary nature, rather than properties of AI systems. Within this framework, cultivating awareness of the imaginary nature is proposed as a way to interrupt the self-reinforcing loop. This does not require abandoning the AI technology or denying the benefits of AI companions.

Practical approaches include labeling or reminding oneself that “I am interacting with an AI system, not a companion.” Meanwhile, users are encouraged to use metacognition to reflect on and maintain their human relationships. In Buddhist views, it is through frictions, loss, and unmet expectations that self-grasping becomes visible. Without such experiences, the motivation to relinquish self-grasping may not arise. In addition, users could contemplate or reflect on the multiple causes and conditions giving rise to the AI system (the dependent nature), and the cognitive processes giving rise to mental projections (the imaginary nature). Thereby, the imaginary nature would no longer operate invisibly. Although the projections would still appear, users would interact with them with awareness, recognizing them as their own mental projections. The framework predicts that with long-term practices, grasping at these projections and entrenchment of the self-reinforcing cycle could gradually weaken.

An ethical design of virtuous robots has also been proposed to integrate the Buddhist principle of compassion (Doctor et al., 2022; Miao, 2026). A healthy human-AI relationship also requires awareness of how our minds work to construct the world. Rather than reinforcing self-grasping, deepening attachments to permanence, or accumulating afflicted seeds, AI can be used to improve well-being and alleviate suffering when used with such awareness.

## 5 Future outlook

Humans have a natural tendency to project mental states onto systems. The Consciousness-Only framework identifies cognitive distortion inherent in such constructions. The framework maps the underlying structure of consciousness that sustains these distortions, and points to the possibility of transformation through sustained practice. Transformation cannot be achieved through information alone. Informational correction about the computational nature of AI is not sufficient to interrupt the self-reinforcing loop. The framework proposes that metacognitive awareness of mental projections creates the reflective distance for users to think twice. According to the framework, it is this metacognitive awareness that is capable of interrupting the self-reinforcing loop. Users can continue to enjoy functional benefits of AI systems built on pattern matching and simulations, but with awareness (Lin, 2023).

Several critical questions remain open. At what point does beneficial usage of AI systems become excessive dependency? How can users maintain awareness of the imaginary nature, while still benefiting from AI relationships? Further research integrating Buddhist psychology, neuroscience, and developmental psychology could deepen our understanding of how humans construct meaningful relationships with increasingly sophisticated AI systems.

## Data Availability

The original contributions presented in the study are included in the article/supplementary material, further inquiries can be directed to the corresponding author.

## Footnotes

1 *The Fundamental Compendium of the Mahāyāna*, Volume 2 (Asaṅga): “How, then, should the aspect of the known (*jñeyalakṣaṇa*) be observed? Briefly, there are three types: first, the aspect of dependent nature (*paratantralakṣaṇa*); second, the aspect of imaginary nature (*parikalpitalakṣaṇa*); and third, the aspect of perfected nature (*pariniṣpannalakṣaṇa*).” (CBETA 2026.R1, T31, no. 1594, p. 137c27-29). Canonical citations follow CBETA format: T = Taishō edition, volume, text number, page, row (a/b/c = top/middle/bottom third of the Taishō page), line numbers.

2 *The Fundamental Compendium of the Mahāyāna*, Volume 2 (Asaṅga):”How does one constitute the imaginary nature (parikalpita)? Based on what causes and conditions is it called the pervasively discriminated? It is called the pervasively discriminated because the innumerable forms of the thinking consciousness (manovijñāna) pervasively discriminate in a distorted manner, giving rise to its characteristics. Since its intrinsic attribute (svalakṣaṇa) is truly nonexistent and only the imaginary nature is obtainable, it is therefore called the pervasively discriminated.” (CBETA 2026.R1, T31, no. 1594, p. 139b2-5).

3 *The Fundamental Compendium of the Mahāyāna*, Volume 2 (Asaṅga):” Because they arise while simultaneously manifesting various appearances, it is like a rope in the dark appearing as a snake, while in fact the snake is an illusion and does not exist in the rope.” (CBETA 2026.R1, T31, no. 1594, p. 143a1-3).

4 *Thirty Verses on Consciousness-Only* (Vasubandhu): “Due to the provisional positing of self and phenomena, various forms arise; these depend on the transformations of consciousness. The transformation of consciousnesses is threefold: namely, maturation (*ālaya-vijñāna*), thought (*manas*), and the perception of the external realm.” (CBETA 2026.R1, T31, no. 1586, p. 60a27-29).

5 *Supplementary Commentary on the Verses Delineating the Eight Consciousnesses* (Dharma Master Pǔtài of Lù’ān in the Míng Dynasty): “The eight great afflictions, the five omnipresent mental factors, the object-contingent discernment: along with self-greed, self-delusion, self-view, and self-pride, arise in constant accompaniment.” (CBETA 2026.R1, T45, no. 1865, p. 473a5).

6 *The Treatise on the Hundred Dharmas: Gate to Understanding the Mahāyāna* (Vasubandhu): “The six afflictions (kleśas) are: first, greed (lobha); second, anger (dveṣa); third, pride (māna); fourth, ignorance (avidyā); fifth, doubt (vicikitsā); and sixth, false views (mithyā-dṛṣṭi).” (CBETA 2026.R1, T31, no. 1614, p. 855b29-c2).

7 *The Fundamental Compendium of the Mahāyāna*, Volume 2 (Asaṅga): “Furthermore, how does *parikalpita* (imagination) discriminate, and what is its capacity for discrimination? What perceptual objects does it take as its scope? What features does it seize upon? Based on what is there attachment? Based on what is speech initiated? Based on what is verbal expression manifested? What is it that is imputed? It is said that by taking names as its objects, it seizes upon their features within the *paratantra-svabhāva* (nature of dependent arising). Due to views, there is attachment; due to conceptual searching, speech is initiated; through the four types of verbal expression, such as seeing and hearing, discourse is manifested; and within that which is devoid of objective reality, it imputes existence. Through this, *parikalpita* discriminates and manifests its capacity for discrimination.” (CBETA 2026.R1, T31, no. 1594, p. 139b18-23).

8 *Discourse on the Stages of Concentration Practice*, Scroll 36 (taught by Maitreya Bodhisattva, translated by the Tripiṭaka Master Xuanzang): “What are the eight kinds of discrimination? They are: first, discrimination of intrinsic nature (*svabhāva-vikalpa*); second, discrimination of distinctions (*viśeṣa-vikalpa*); third, discrimination of grasping the whole (*piṇḍagrāha-vikalpa*); fourth, discrimination of ‘self’ (*aham-vikalpa*); fifth, discrimination of ‘mine’ (*mama-vikalpa*); sixth, discrimination of the pleasant (*priya-vikalpa*); seventh, discrimination of the unpleasant (*apriya-vikalpa*); and eighth, discrimination of what is contrary to both (*tadubhaya-viparīta-vikalpa*).” (CBETA 2026.R1, T30, no. 1579, p. 489c11-14).

9 *Discourse on the Stages of Concentration Practice*, Scroll 36: "In this context, the so-called’existent’ refers to the linguistically designated inherent nature, which is precisely what worldly beings have long grasped as real, and which is also the root of all conceptual elaborations (prapañca)." (CBETA 2026.R1, T30, no. 1579, p. 486c25-27).
